# Lactopeptine

**Published:** 1878-09

**Authors:** 


### Lactopeptine.

In the summer diarrhœas of children we have found *Lactopeptine* of the very highest value. It is probable that weakening of the digestive powers is a very important factor in the causation of Cholera Infantum. We have found *Lactopeptine* a most important help in restoring these cases, when they have passed through the worst stages of that disease, as well as in warding it off when its onset seemed almost inevitable.

In the exhausting Vomiting of Pregnancy, we have found it of very great value in enabling the patient to obtain some nourishment from the food ingested, even if it remained but a short time in the stomach. In the nausea and indigestion and cardialgia, which causes so much annoyance, even if no great danger, in the latter months of gestation, *Lactopeptine* has proved itself almost a specific.

The article used was manufactured by the New York Pharmacal Association.—*St. Louis Clinical Record, July*, 1878.

